# COVID19 Drug Repository: text-mining the literature in search of putative COVID19 therapeutics

**DOI:** 10.1093/nar/gkaa969

**Published:** 2020-11-09

**Authors:** Dmitry Tworowski, Alessandro Gorohovski, Sumit Mukherjee, Gon Carmi, Eliad Levy, Rajesh Detroja, Sunanda Biswas Mukherjee, Milana Frenkel-Morgenstern

**Affiliations:** Laboratory of Cancer Genomics and Biocomputing of Complex Diseases, Azrieli Faculty of Medicine, Bar-Ilan University, Henrietta Szold 8, Safed 13195, Israel; Laboratory of Cancer Genomics and Biocomputing of Complex Diseases, Azrieli Faculty of Medicine, Bar-Ilan University, Henrietta Szold 8, Safed 13195, Israel; Laboratory of Cancer Genomics and Biocomputing of Complex Diseases, Azrieli Faculty of Medicine, Bar-Ilan University, Henrietta Szold 8, Safed 13195, Israel; Laboratory of Cancer Genomics and Biocomputing of Complex Diseases, Azrieli Faculty of Medicine, Bar-Ilan University, Henrietta Szold 8, Safed 13195, Israel; Laboratory of Cancer Genomics and Biocomputing of Complex Diseases, Azrieli Faculty of Medicine, Bar-Ilan University, Henrietta Szold 8, Safed 13195, Israel; Laboratory of Cancer Genomics and Biocomputing of Complex Diseases, Azrieli Faculty of Medicine, Bar-Ilan University, Henrietta Szold 8, Safed 13195, Israel; Laboratory of Cancer Genomics and Biocomputing of Complex Diseases, Azrieli Faculty of Medicine, Bar-Ilan University, Henrietta Szold 8, Safed 13195, Israel; Laboratory of Cancer Genomics and Biocomputing of Complex Diseases, Azrieli Faculty of Medicine, Bar-Ilan University, Henrietta Szold 8, Safed 13195, Israel

## Abstract

The recent outbreak of COVID-19 has generated an enormous amount of Big Data. To date, the COVID-19 Open Research Dataset (CORD-19), lists ∼130,000 articles from the WHO COVID-19 database, PubMed Central, medRxiv, and bioRxiv, as collected by Semantic Scholar. According to LitCovid (11 August 2020), ∼40,300 COVID19-related articles are currently listed in PubMed. It has been shown in clinical settings that the analysis of past research results and the mining of available data can provide novel opportunities for the successful application of currently approved therapeutics and their combinations for the treatment of conditions caused by a novel SARS-CoV-2 infection. As such, effective responses to the pandemic require the development of efficient applications, methods and algorithms for data navigation, text-mining, clustering, classification, analysis, and reasoning. Thus, our COVID19 Drug Repository represents a modular platform for drug data navigation and analysis, with an emphasis on COVID-19-related information currently being reported. The COVID19 Drug Repository enables users to focus on different levels of complexity, starting from general information about (FDA-) approved drugs, PubMed references, clinical trials, recipes as well as the descriptions of molecular mechanisms of drugs’ action. Our COVID19 drug repository provide a most updated world-wide collection of drugs that has been repurposed for COVID19 treatments around the world.

## INTRODUCTION

The COVID-19 pandemic outbreak has triggered immediate reactions from the medical and scientific communities, and has resulted in an explosive growth of novel data regarding possible therapies or therapeutic opportunities (1,2). The COVID-19 data portal (https://www.covid19dataportal.org/) established by the European Commission in April, 2020 has facilitated the exchange and sharing of COVID-19 research data. One of the first open initiatives realized with creation of this portal was the development of the COVID-19 Open Research Dataset (CORD-19) (2). The CORD-19 (https://www.semanticscholar.org/cord19) currently lists ∼130,000 articles from the WHO COVID-19 database, PubMed Central, medRxiv, and bioRxiv, as collected by Semantic Scholar. Another comprehensive list of COVID-19 databases and journals can be found on the Centres for Disease Control (CDC) library webpage:

https://www.cdc.gov/library/researchguides/2019novelcoronavirus/databasesjournals.html.

According to recent records from LitCovid resource (1), 40 300 COVID19-related articles have been currently listed in PubMed (1). The rapid accumulation of COVID-19 literature requires novel tools for the data collection and organization with efficient navigation capabilities. Such navigation capabilities are based on the literature-based discovery (LBD) concept (3) and can be achieved by implementing text-mining, clustering, and classification methods (1,4–8). Available text and data-mining tools, such as those found at LitCovid (1), PubTator (4,9,10), the iSearch platform (https://icite.od.nih.gov/covid19/search/), NeuralCovidex (https://covidex.ai/) (7), the COVID-19 Data Portal (https://www.covid19dataportal.org/), Carrot/Lingo (https://search.carrot2.org/#/web) (11) and ProtFus (12), efficiently extract target information across articles and other text sources. Using the mentioned tools for text-mining, we have created the COVID-19 Drug Repository.

The goal of our COVID-19 Drug Repository was to automatically collect data on drugs used against COVID-19 around the world and build a structured repository that includes drug descriptions, side effects and available publications. The repository also contains medicine- and pharmacology-oriented data, including annotated information on (FDA-)approved drugs, therapeutic agents (experimental drugs), and drug-like synthetic or natural chemical substances. The data was collected and integrated by methods developed for the ‘omics’ field (13), in particular, chemogenomics (i.e. chemical genomics) (14–17), pharmacogenomics (18–27), genomics and personomics (28–30). In addition, we made use of a number of chemogenomics (31–33) and pharmacogenomics (34–36) approaches that focused on the repositioning (i.e., repurposing) of FDA approved drugs and clinical trials in the treatment of COVID-19. All the data collected in the COVID-19 Drug Repository are designed for use by researchers and clinicians in the field. The information cannot be used for self-medication!

## RESULTS

### The COVID-19 Drug Repository: structure and technical description

**Figure 1.**
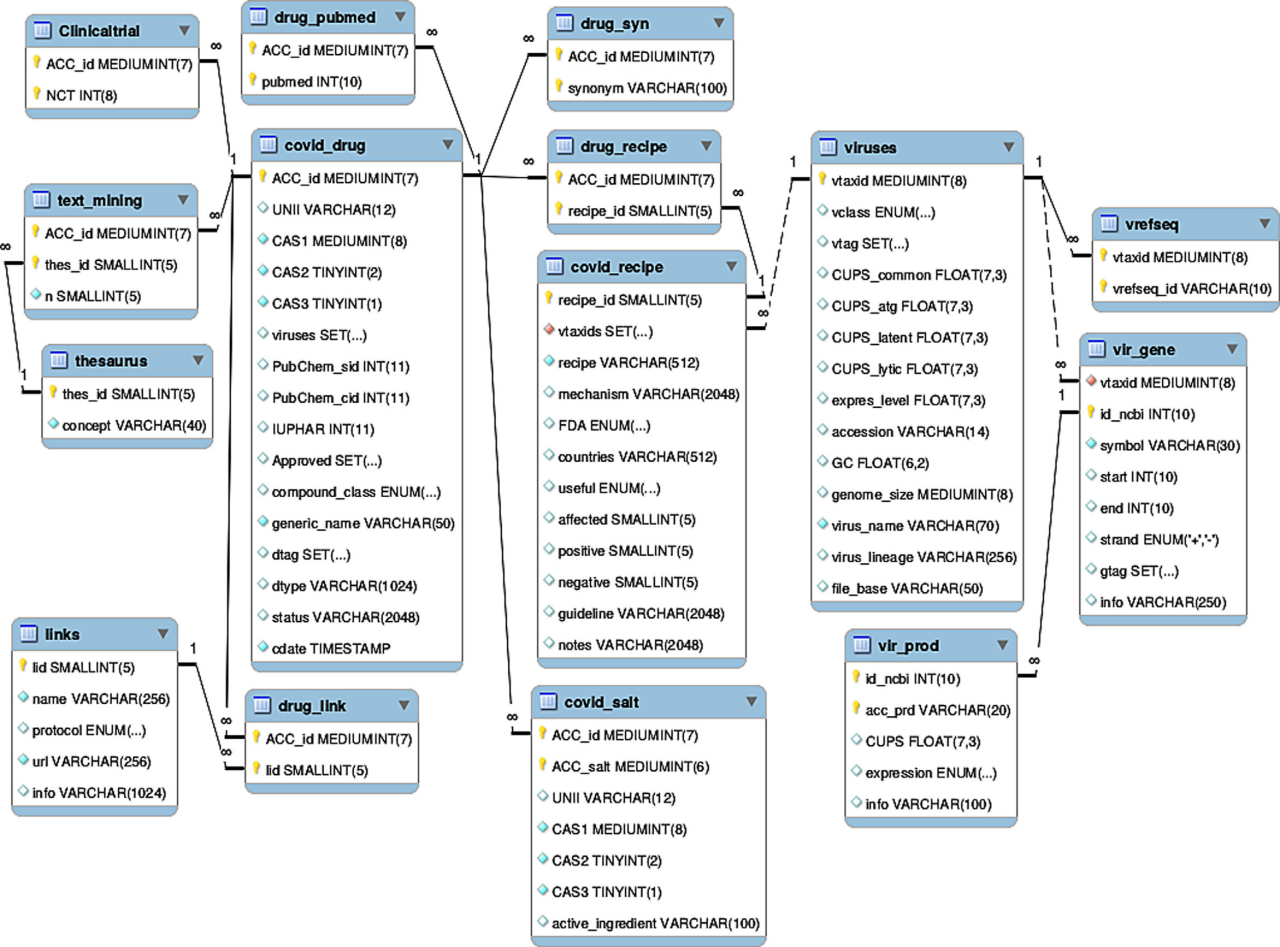
The structure/map of the COVID-19 Drugs Repository.

**Figure 2.**
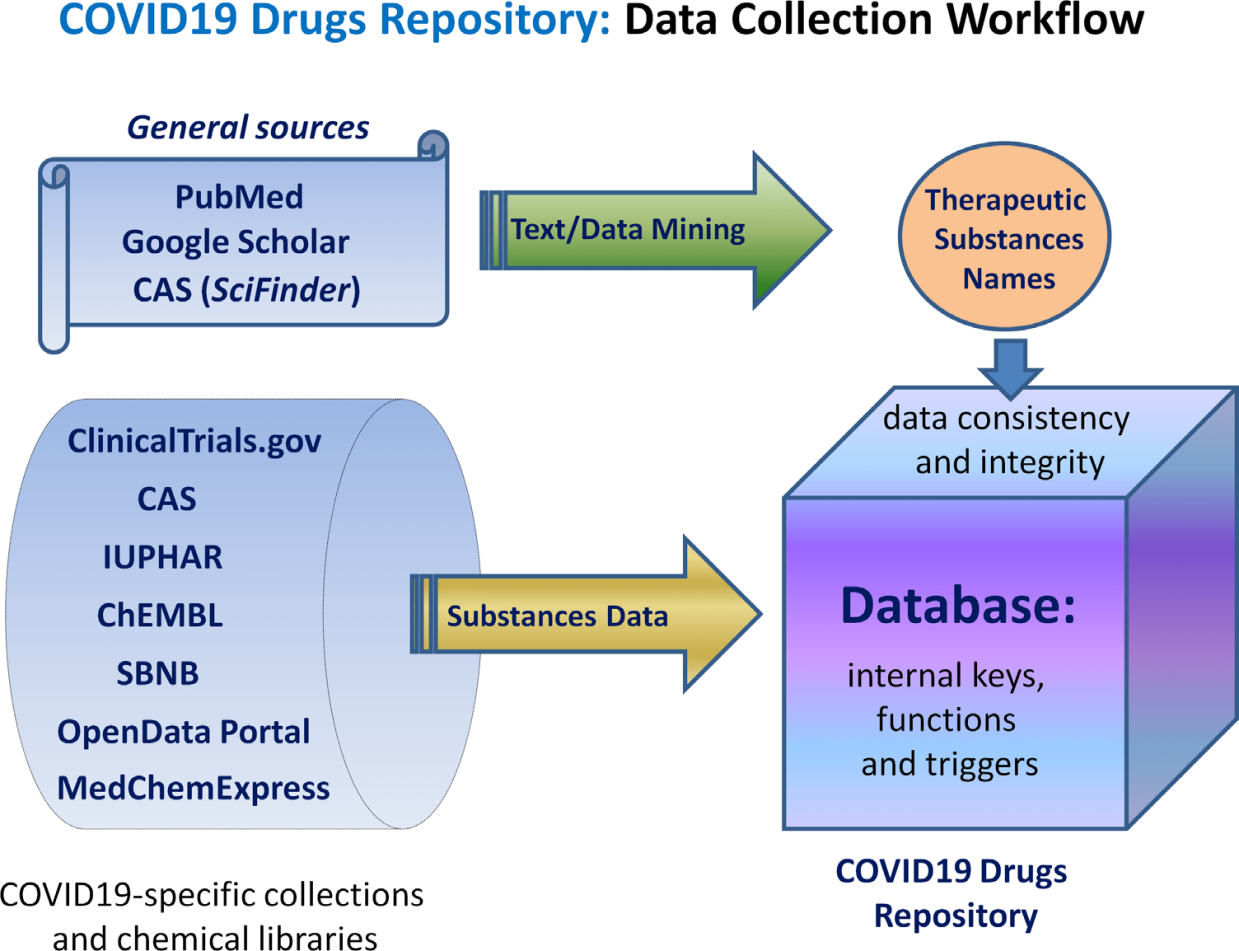
The data collection workflow implemented in the COVID-19 Drugs Repository.

**Figure 3.**
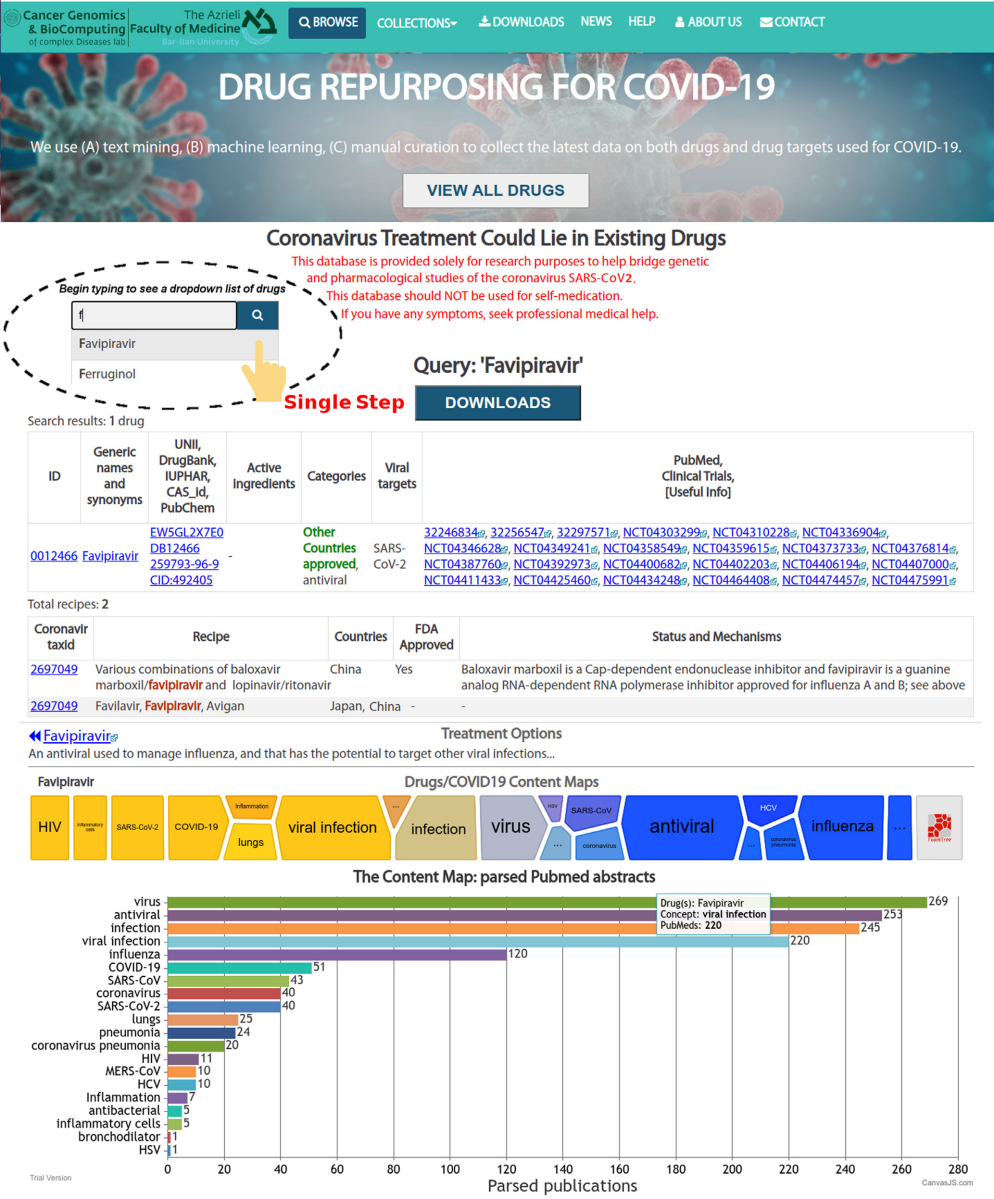
Web interface of the COVID19 Drug Repository. Selection of the drug of interest (e.g. *Favipiravir*) from the dropdown menu/list.

COVID-19 Drug Repository is an open-source modular platform built on the MySQL server platform, comprising 15 curated tables. The structure of the database, presenting the logical relations between these tables, and the data collection process are shown in Figures 1 and 2, respectively. To ensure consistency between the *drug_syn*, *drug_recipe*, *covid_salt*, *drug_link*, *drug_pubmed*, *Clinicaltrial*, *text_mining* and *covid_drug* tables, the insertion/update/deletion of rows is linked to the *covid_drug* table. Each *covid_drug* entry is linked to 15 data fields corresponding to drug data and a target. Most of the data fields (i.e. ACC_id, UNII, CAS1, CAS2, CAS3, and PubChem_cid) are hyperlinked to other databases (i.e. DrugBank (37,38), ClinicalTrials.gov, PubChem (39), IUPHAR/BPC (40) and Chemical Abstracts Service (41,42) (Figure 1, Figure 2, Table 1). Each *covid_recipe* entry is associated with 12 data fields, including drug formulation (recipe), citing the country of manufacture, FDA-approved drugs, guidelines, etc. The COVID-19 Drug Repository supports text query inputs using the search box on the homepage (Figure 3). The MyISAM engine (43) was implemented to support the FULLTEXT search functionality, with the ‘utf8’ DEFAULT CHARSET. Detailed instructions on the browsing and search tools found in the database were provided below and can also be found on the database homepage (under ‘Help’ option). Finally, the database update process is semi-automatic as follows: (a) selection of potential COVID19 therapeutic substances found in research articles is manual; (b) updating and adding new records to COVID19 Drug Repository database is fully automated using Perl scripts; (c) hyperlinks to PubMed and other sources, and maps are generated automatically by python scripts.

**Table 1.** Databases linked with the COVID-19 Drugs Repository

Database Identifier	Database/resource	Links
ACC_id (*)	COVID19 Drug Repository	http://covid19.md.biu.ac.il/
UNII ID	Unique Ingredient Identifier	https://www.fda.gov/industry/fda-resources-data-standards/fdas-global-substance-registration-system
CAS ID (**)	Chemical Abstracts Service	https://www.cas.org/
PubChem CID	PubChem Compound	https://pubchem.ncbi.nlm.nih.gov/
PubChem SID	PubChem Substance	https://pubchem.ncbi.nlm.nih.gov/
PubMed ID	PubMed	https://pubmed.ncbi.nlm.nih.gov/
DrugBank ID	DrugBank	https://www.drugbank.ca/
IUPHAR ID	IUPHAR/BPS	https://iuphar.org/
Clinical Trials ID	ClinicalTrials.gov	https://clinicaltrials.gov/

### The database search query syntax

The database search is case-insensitive. Simple queries can include either the full or partial drug name (‘Drug search’ box). Advanced queries can be constructed by combining identifiers of the various databases (Table 1) and/or concepts (e.g. compound class, viral target, etc.). The ‘compound class’, for example, includes the following terms: «Antibody | Metabolite | Natural product | Inorganic | Peptide | Synthetic organic», while the ‘viral target’ category comprises the «acronym of viral name»: «BCV | BtCoV-(HKU3 | HKU5 | SHC014) | HcoV-(229E | NL63 | OC43) | (MERS|SARS)-CoV(-2)? | CcoV | FIPV | PEDV | SARS | TGEV | IBV | MHV». A query combination can be built using delimiters « and », « plus », « + », «[]&[]», «[];[]» (the «[]» indicates that the ‘space’ delimiter may be used in the query string), for example:

*Query*: Oseltamivir; Ritonavir; DB0008934; PMID32145363; CID:37542; 118390–30-0

The search results page shows all relevant instances associated with a query drug. The HTML page is generated with hyperlinks to all databases (Table 1) associated with the drug of interest. Alternatively, the information can also be accessed by selecting a drug name from the menu in the selection field (see the Database ‘Help’ page: http://covid19.md.biu.ac.il/). The «Treatment Options» section enables access to a detailed description of the query drug.

### COVID19 Drug Repository web interface

On the menu bar, there are seven buttons (BROWSE, COLLECTIONS, DOWNLOADS, NEWS, HELP, ABOUT US and CONTACT on the right and the ‘Cancer Genomics & BioComputing of complex Diseases lab’ logo on the left) that users can visit with a single click. Clicking the BROWSE button returns the user to the homepage (Figure 3), which displays introductory and technical information about the Repository. Clicking the ‘VIEW ALL DRUGS’ button or on the ‘COLLECTIONS’ submenu items (e.g. ‘Anti-viral drugs’) brings the user to the drugs-listing webpage (Figure 3), where one can see all query drugs presented there. By entering the first letters of a query drug name in the ‘Drug search’ field (Figure 3), users can select their drug of interest from the dropdown menu (Figure 3).

### Features and functionality

The Repository is a COVID19-targeted collection (short-list) of ∼460 items representing 184 approved drugs, 384 investigated therapeutic agents and 76 drug-like synthetic or natural chemical substances. The main focus of the repository is cross-referencing to PubMed articles linking these drugs with multiple research sources, mapping associations between the drugs and COVID19-related concepts, text/data-mining, clustering, and visualization. Furthermore, the Biopython collection of modules (44), in particular, the Entrez Bio.Entrez module (44), was implemented into our database framework so as to enable fast data retrieval via efficient command-line interactions with all NCBI resources and databases (45), sub-divided into six categories: Literature, Genes, Proteins, Genomes, Genetics, and Chemicals. A toolset of Perl and Python scripts had been created for specific tasks, such as (a) automatic generation of links between the Repository and other sources, (b) collection of data/references and creation of work tables and (c) mapping associations/concepts, with visualization options being realized via external tools.

### COVID19 drugs: search strategy

Recent information on approved drugs and therapeutic combinations thereof considered useful for the treatment of SARS-CoV-2 infection has been reported at ClinicalTrials.gov and PubMed. Experimental therapeutic agents, drug-like synthetic or natural chemical substances have also been studied in the context of COVID-19, as reported in the PubMed database. To extend the coverage of our database, we reviewed articles from PubMed to extract COVID-19-related concepts and keywords. Specifically, using ‘drug repositioning’, ‘drug repurposing’, ‘COVID19’ and ‘coronavirus infections/drug therapy’ as query keywords, we reached PubMed publications with titles and/or abstracts containing combinations of these keywords. Next, we created another pattern of queries to search target information at PubMed, and using Google and Google Scholar. The pattern was expressed as a pseudo-Regular Expression (46), where text in uppercase letters denotes variable names:

‘((DRUG_NAME)|(DRUG_ALIAS)) ((sars)|(mers)|(corona covid)) (patients)?’

Examples of the multi-step search queries are as follows:

‘camostat corona covid’ → [output_1: information/references] → [therapeutic agents]

‘camostat sars’ → [output_2: information/references] → [therapeutic agents]

‘camostat sars patients’ → [output_3: information/references] → [therapeutic agents]

‘ONO-3403 sars patients’ → [output_4: information/references] → [therapeutic agents]

Using this search strategy, we found ∼100 substances with activities associated with COVID-19. The queries and the patterns used, and the information obtained daily are the main sources for Repository updates.

All ‘active’ chemical entities/substances (i.e. those with demonstrated or proposed therapeutic potential) were collected and linked via their identifiers in CAS, PubChem, IUPHAR, etc. Furthermore, we adopted the web-based text clustering engine Carrot^2^ (47) for visualization of pair-wise ‘drug-COVID-19 concept’ associations found in PubMed abstracts for each pair.

In this version of our database, COVID19 drugs were mapped to a dictionary of 21 terms related to concepts of ‘COVID-19’, e.g. ‘viral infections’, ‘respiratory diseases’, ‘inflammatory cell’, ‘coronavirus pneumonia’, etc. (Supplemental Table S1). These terms are the most frequent words/combinations clustered around the central words such as ‘virus’, ‘infection’, ‘inflammation’, ‘pneumonia’, ‘lungs’. To create the concepts’ dictionary, a variety of clusters were generated by experimentation with different hierarchical clustering algorithms applied to the collection of PubMed titles/abstracts. Links to all PubMed abstracts associated with these ‘drug-concept’ pairs were generated and enumerated using Python scripts. PubMed search queries were created according to PubMed query syntax (48) and MeSH terms (49). The automatically generated tables (available in the ‘DOWNLOADS’ section) list the number of retrieved PubMed publications corresponding to each ‘drug-COVID-19 concept’ pair. These numbers are hyperlinked with the corresponding PubMed publications. Links to references and the Carrot^2^ text clustering and visualization tool can be updated on a regular basis. Such updates are necessary as the web and PubMed database are constantly expanding, with new references and sites appearing daily. All desired data can be downloaded from the COVID19 Drug Repository website (link) as Excel tables containing the list of keywords (i.e., the ‘dictionary’) used for text-mining and mapping. In subsequent versions of the database, users will be able to modify the list or introduce additional concepts. With this simple mapping tool, one can discover and visualize new concepts and associations that would not otherwise be found.

### Drug repurposing sources

Currently, there are 384 mapped drug names mentioned in 960 COVID-19 clinical studies (data retrieved on August 15, 2020), with at least 1 drug intervention (Table 2). None of these drugs are novel. Rather, they exemplify a ‘drug repurposing/repositioning’ approach (26,34,50–52). Recently, numerous COVID19-specific web pages and chemical libraries have been created by different research organizations (CAS, IUPHAR (53,54), ChEMBL, OpenData Portal (https://opendata.ncats.nih.gov/covid19/index.html), etc.) and companies (MedChem Express), and used for the high throughput screening against SARS-CoV-2 infection (55–63). All these molecular libraries and collections (Figure 2, Table 2) are being used in our data collection process (Figure 2), and listed in the Repository web page (‘Useful Links’).

**Table 2.** COVID19-specific collections and chemical libraries

Resource	Dataset (title, catalogue, or category)	Drugs, active agents	Links
ClinicalTrials.gov	COVID-19 studies by mapped drug intervention	384	https://clinicaltrials.gov/ct2/covid_view/drugs
CAS	COVID-19 Antiviral Candidate Compounds Dataset	∼50,000	https://www.cas.org/covid-19-antiviral-compounds-dataset
IUPHAR	Ligands relevant to SARS-CoV-2(COVID-19	64 items	https://www.guidetopharmacology.org/GRAC/CoronavirusForward
ChEMBL	ChEMBL_27 SARS CoV-2 Release, 8 datasets	133 selected (IC50/EC50 better than 10 μM)	http://chembl.blogspot.com/2020/05/chembl27-sars-cov-2-release.html
Structural Bioinformatics and Network Biology Group (SBNB)	Drugs from sthe COVID19 literature	307	https://sbnb.irbbarcelona.org/covid19/
OpenData Portal	NCATS Anti-infectives Collection	740	https://opendata.ncats.nih.gov/covid19/index.html
MedChemExpress	SARS-CoV List of Drugs	76	https://www.medchemexpress.com/Targets/SARS-CoV.html
MedChemExpress	Anti-Virus Compound Library	512	https://www.medchemexpress.com/screening/Anti-virus_Compound_Library.html
MedChemExpress	Anti-COVID-19 Compound Library	1552	https://www.medchemexpress.com/screening/anti-covid-19-compound-library.html

### Drug-gene interaction networks

**Figure 4.**
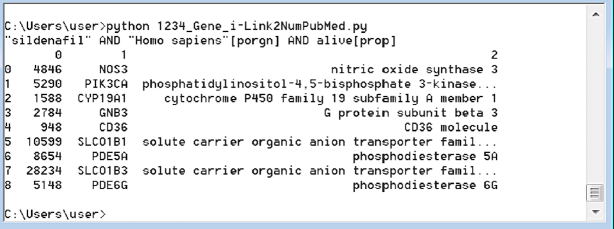
Example output of the ***DruGeNetwork*** python script: list of genes (column ‘1’) with a short description/annotation of the underlying biological mechanism associated with each gene (column ‘1’). The output is generated for the query keywords sent to the NCBI Gene resources: “sildenafil” AND “Homo sapiens”[porgn] AND alive[prop].

The Biopython/Entrez-based Python command-line script (as discussed in the Features and functionality section) was created to access the NCBI Gene database (45), and to automatically retrieve human or microbial (and in particular, viral) genes associated with a given list of drugs or chemical substances. The output (Figure 4) provides a list of genes with a short description of the biological role associated with each gene product in the output list.

Those genes associated with a set of drugs can be analysed, clustered, or served as input for building ‘drug-gene’ networks and then visualized using external programs. As a working example, protein-protein association networks were built for output gene sets using the STRINGv11 database (64). Moreover, the application programming interface (API) implemented in the STRINGv11 database enables efficient interaction of external databases with the STRING visualization and analysis tools (64). For example, visualization of the set of genes associated with the PDE5A inhibitor sildenafil, a vasodilator agent, revealed other interesting targets (Figure 4), such as the enzyme PDE6G. Both enzymes are active in the lungs (65,66). Further network analysis of available data showed that PDE5A/PDE6G inhibition by sildenafil in lung blood vessels can trigger different anti-inflammatory pathways.

**Figure 5.**
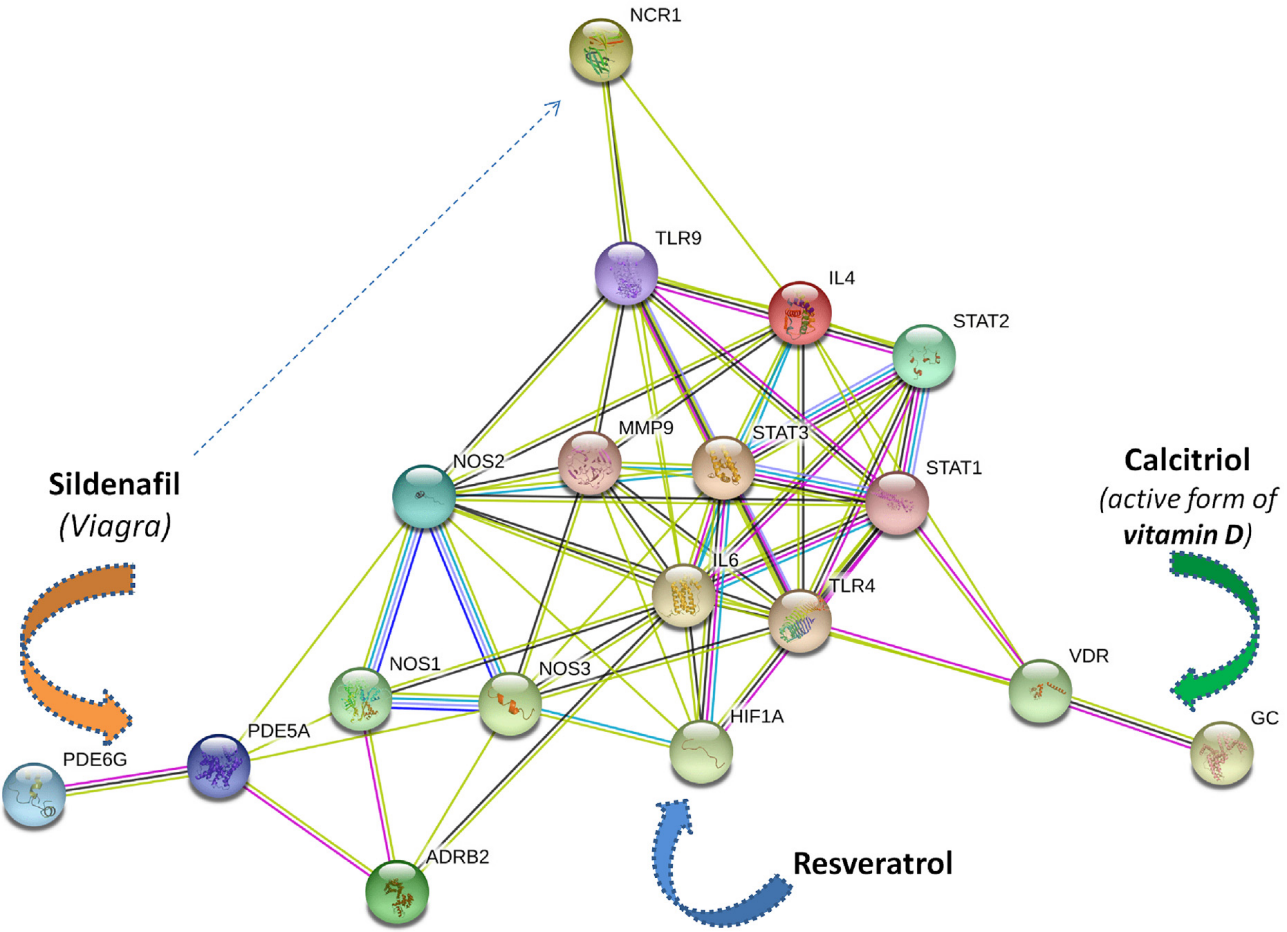
The Drug-Gene local Network built using the output for sildenafil (**A**). Further functional enrichment reveals the group of genes involved in the innate immunity and inflammatory processes. Moreover, VDR (receptors activated by calcitriol), and the HIF1A-STAT3 path suppressed by resveratrol, are also involved in the local anti-inflammatory pharmacological network, thereby suggesting the “sildenafil-resveratrol-vitamin D3” drug combination to treat the COVID-19 complications

To build a network (Figure 5), we extracted additional information from the literature and external databases. In the PDE5A and PDE6G protein expression summaries obtained from the Human Protein Atlas (67), the PGE6G gene is categorized as ‘Group enriched’ in natural killer (NK) cells, according to consensus transcriptomics data. NK cells, acting as cytotoxic lymphocytes, are involved in innate immune system regulation, including rapid cytokine production in the presence of virus-infected cells (67). The NK-mediated antiviral immune response is associated with the NCR1 gene that encodes the natural cytotoxicity receptor 1 (68). In the next step, both the PDE6 and NCR1 genes were detected in the Chronic Obstructive Pulmonary Disease (COPD)-related Gene Set using Harmonizome on the collection of ‘omics’ Big Data sets (69). This gene set was deposited in the GEO Signatures of Differentially Expressed Genes for Diseases, under the name ‘COPD-Chronic Obstructive Pulmonary Disease_Muscle-Striated (Skeletal)-Diaphragm (MMHCC)_GSE47’. The data show that the expression of the PDE6G gene is significantly increased, whereas decreased expression was reported for the NCR1 gene.

Therefore, in the context of drug repurposing strategies, it is reasonable to expect that sildenafil will be useful for the treatment of COVID19 complications. Accordingly, two recent clinical studies (ClinicalTrials.gov identifiers NCT04304313 and NCT04489446) were initiated to study the efficacy and safety of sildenafil in patients with COVID-19 (NCT04304313), and to assess the role of sildenafil in improving oxygenation among hospitalised patients (NCT04489446).

### Identification of drug target genes

We extracted target gene information for each putative COVID-19 drug from the Therapeutic Target Database (70), and by text-mining of the literature at PubMed. To understand the expression profile of drug target genes identified in this manner in the COVID-19 infection, we performed transcriptome analysis of infected bronchial epithelial cells. For this, we retrieved raw RNA-sequencing data for SARS-CoV-2-infected bronchial epithelial cells from the sequence read archive (SRA) database under accession no. PRJNA615032 (71). The FASTQ files were mapped and aligned to the hg38 reference genome using STAR (72). Differentially expressed genes were identified using edgeR (73), with parameters set at 2.0-fold change and <0.05 *P*-value cut-off. We thus found target genes for 41 drugs from our database (e.g. sildenafil as discussed in previous paragraph) which are significantly differentially expressed during COVID-19 infection. These drug-gene pairs are given in the Supplemental Table S2.

### COVID19 Drug Repository server, hardware and software requirements

The COVID19 Drug Repository server was built on an Apache web server and deployed on the RedHat Enterprise Linux (RHEL) 7.4 server of an Intel(R) Xeon(R) CPU E5–2620 v2 @ 2.10GHz and 32GB RAM unit. The COVID19 Drug Repository has a code base and infrastructure similar to that of the ChiTaRS database (74,75). The COVID19 Drug Repository website is compatible with modern web browsers (such as Chrome, Firefox, Microsoft Edge, Opera and Safari), provided that JavaScript is enabled. We recommend using the latest release version of these web browsers for optimal rendering.

### Downloads

The COVID19 Drug Repository not only provides extended ‘Search’ options but also offers the possibility to download all database tables and data sets in a user-friendly manner. The repository is available at: http://covid19.md.biu.ac.il/.

## CONCLUSIONS AND FUTURE PLANS

The COVID19 Drug Repository maps data from chemogenomics and pharmacogenomics studies and provides viral and human genomics and proteomics information on approved drugs and other therapeutics. The database enables the user to focus on different levels of complexity, starting from general information, clinical trials and formulations, and increasing the resolution to the level of molecular mechanisms of drug action. Therefore, the database can serve as a navigation and recommendation tool both for research and for healthcare purposes. Future plans include the following additions to the database: (a) continuous updating with new data on approved drugs, experimental drugs, and drug-like synthetic or natural chemical substances; (b) automatic machine learning and text-mining-based annotation and visualization of ‘Mode of Action’ (MoA) data, as well as ‘Drug-Gene’, and ‘Drug-Symptom’ networks; and (c) incorporation of the Drugs/NGS analysis tools (‘transcriptomics’) to accelerate the translation of knowledge for use as personalized medicine for COVID19 patients.

## DATA AVAILABILITY

http://covid19.md.biu.ac.il/.

## Supplementary Material

gkaa969_Supplemental_Files
